# Typhoid Fever, with Report of Cases

**Published:** 1900-06

**Authors:** W. B. Hardman

**Affiliations:** Harmony Grove, Ga


### TYPHOID FEVER, WITH REPORT OF CASES.*

By W. B. HARDMAN, M.D.,

HARMONY GROVE, GA.

I availed myself of the opportunity at the last meeting of the Georgia Medical Association to make a few remarks concerning the

*Read before the Georgia Medical Association, April, 1900.

eliminative and antiseptic or Woodbridge treatment of typhoid fever. Since that meeting I have had some experience along this line, which, added to my previous experience with this method of treating typhoid, has given me great confidence in it.

I am aware that the treatment of typhoid fever is an old subject, and the discussion of it may bore some of my hearers. But I would ask: While it is old is there any subject in medicine equally as ancient concerning which there is so much difference of opinion? Or is there any disease known to medicine of equal importance to the general practitioner? One that has come in mortal combat with as near all of the pharmacopeia? It has met on the arena almost every remedy from cold water to coal-tar, from gelsemium to guaiacol, and from calomel to constipation, "and has never yet lowered its arm."

It is said by many to be a self-limited disease, but unfortunately in many instances it holds a better limit than the patient.

It is not an uncommon occurrence for certain physicians in every community to get a reputation for being "good fever doctors," and all of these "good fever doctors," perhaps, have certain pet methods of treating typhoid which they consider just a little better than any other method, and yet every one of them may go at it differently. Now this argues one of two things. It either shows that almost anything will do to use in treating typhoid, or, so far, no method has been discovered which proves anything like universally satisfactory.

As for my part I believe that the use of eliminatives and antiseptics combined have marked an era in the treatment of typhoid. Subsequent experience may give me reason to change my mind. If so, I am not so thoroughly biased that I cannot do so. I have no motive whatever in championing the cause of Dr. Woodbridge. He is no more to me than any other man; but I thank him for his suggestions, and I believe him to be a benefactor to the race. I believe his method, or modification of his method, of treating typhoid is the safest, surest, shortest, the most agreeable, rational and universally practicable method now extant.

The Brand treatment, which no doubt answers well in some cases and in some places, is totally impracticable and unsuited for general use. How could the country practitioner carry it out with half a dozen cases on his hands at the same time, several miles apart, and all of them to be furnished with a suitable bath tub for taking a plunge bath every three hours. In a city or village outside of a regular hospital the same objection applies, though perhaps not in such a marked degree. To the majority of physicians the Brand treatment is out of the question, for it would be about as reasonable for them to order their patients to take a yacht ride three times a day as a plunge bath every three hours according to the direction of Brand. I for one do not believe that the human family is greatly the loser on this account.

The modifications of the Brand treatment, such as cold sponging or stripping the patient and using cold water or ice with massage, are very troublesome and clumsy procedures if properly and faithfully administered. Nine times out of ten the modifications of the Brand treatment are not thorough or systematic enough to be of great benefit in shortening the course of the disease. They sometimes render the patient more comfortable.

The general use of the coal-tar preparations is dangerous.

The use of mineral acids, nitrate of silver, turpentine, iodine, etc., I believe is some better than doing nothing at all.

The expectant plan, the do-nothing, the do-everything, and the constipation methods are about on a parity. The physicians who adopt these methods are the doctors who usually get the reputation of being "good fever doctors." *They are good nurses.* They see their patients often and long, and manage to keep them sick from six to eight weeks; but finally pull them through by the "skin of their teeth," or rather pull them through only "skin and teeth." They get for their *scientific treatment* a fat fee and the thanks of their patients for their careful attention and nursing.

I do not think that I am hasty in forming opinions, or that I become overzealous in advocating anything until I have pretty fair reasons for so doing. What I say now in reference to the Woodbridge treatment of typhoid I leave open to modification when experience teaches me differently. I am not so captivated that I will not listen to reasonable arguments against it, but I have little patience with the doctors who oppose it on the grounds that because the patients get well in from ten to fourteen days they doubt the diagnosis. Delafield, Pepper and many distinguished authors speak of the abortive form of typhoid which naturally gets well in ten to fourteen days. Delafield also says that there is usually a remission of temperature at the end of the second week in typhoid, with an apparent tendency to convalescence. Why not with proper treatment make all the cases the abortive type? I am almost persuaded to say that if the treatment is begun early enough and properly administered it can be done.

Much has been said about mistakes in diagnosis in these quickly cured cases. Dr. Osler, who has little faith in the Woodbridge treatment, says that the physicians should suspect typhoid fever in every case of continued fever of six or seven days' duration, especially if it resisted quinine, and he lays stress on the fact that the physician very often calls a typhoid fever malaria, rather than the reverse. My experience bears out this observation. I mention this to show that the physician who has many cases of fever of from six to fourteen days' standing must surely have some typhoid among them. Simply because his fever cases never run longer is no proof that he has no typhoid.

I began the use of the Woodbridge treatment exclusively in all the cases that I diagnosed to be typhoid fever five years ago the first of this month, and only in two cases have I seen an elevation of temperature after the fourteenth day. One of these was a boy of twelve who had a febrile movement for twenty-five days, but for more than a week after the diagnosis was made he was not put upon the Woodbridge tablets because he persistently vomited them. This is the only case I have met with which could not take them well, or offered any serious objection to taking them. The other case where there was an elevation of temperature after the fourteenth day will be mentioned later.

I want to give you a short record of sixteen cases treated since the last meeting of the Association. Out of these sixteen cases I had two deaths, but after having a record of these two cases I am inclined to think you will not care to count them against the record of the Woodbridge treatment.

I will group my cases somewhat geographically, as this will furnish some strong circumstantial evidence in confirming the diagnosis. In none of these cases did I stain for the bacillus of Eberth, but all of the symptoms and surrounding circumstances make the diagnosis of all but one or two of the cases conclusive.

The first four cases, all white females, lived at a place, and adjoining a place, where, two years previous, were some severe and protracted cases of typhoid.

1. Mrs. W., aged 30, was sick four days before calling me. Had taken calomel and quinine but gradually grew worse. Diagnosis at first call. Developed to be as well-marked case of typhoid as I ever attended. Dry furred tongue, tympanitic bowel, nose bleed, temperature 103 morning, 105 afternoon. She was an exceedingly sick woman for the time. Temperature normal on fourteenth day of treatment, and with the exception of almost a general neuritis which developed during the second week of her illness, she made a good recovery.

2. Hattie W., aged 10, daughter of Mrs. W., developed typical typhoid. Temperature normal on the tenth day of treatment.

3. Mrs. D., aged 45, neighbor to Mrs. W., sat up with and helped nurse her during her illness. Developed typical typhoid. Temperature normal on thirteenth day of treatment.

4. Miss D., aged 15, only child of Mrs. D., was in constant attendance upon her mother. Developed typhoid soon after her mother's recovery. Temperature normal on the eleventh day of treatment.

5. Mrs. W., again, aged 19, newly married, visited her parents who lived near mill-pond and creek. Found two brothers at home with chills and fever. Remained a week. Returned home feeling badly and gradually continued to grow worse for eight or ten days before calling me in. She kept up most of the time, making little complaint as her husband was night policeman and slept during the day. When I called found temperature 105; no special tympanitis, complained of aching all over, was in a moist perspiration, tongue red with some coating. Got her history and from general appearance concluded that she had malarial remittent fever. Gave calomel and quinine. Next day no improvement. Increased quinine. Began arsenic and muriatic acid and phenacetin when needed to reduce temperature. The following day I found no decided improvement and began to suspect typhoid, though there was no tympanitis and but little tenderness in the bowel. Her bowels were inclined to constipation when left alone. I left off quinine and muriatic acid and began at once with No. 2 Woodbridge tablets every half hour. These were continued about thirty-six hours in all, though not so frequently all of this time, when she had a violent hemorrhage. Discontinued Woodbridge altogether, principally because disease was too far advanced for the treatment to be of much benefit. Directed my attention principally to the treatment of the hemorrhage, but in spite of all medication the hemorrhage was repeated every afternoon for four days, when she died.

Some may claim that the Woodbridge tablets caused the hemorrhage in this case, but I do not believe it. This patient insisted on getting up to the mug for her bowels to move or even to void her urine from the beginning of her illness until the hemorrhage occurred. This may have helped to bring it about.

The only other case of serious hemorrhage I ever had was in a woman of constipated bowels, whom I was treating for supposed malaria, because her symptoms and surroundings were malarial, and her husband was just recovering from regular chills and fever. I put her on the Woodbridge treatment and her temperature was normal in two weeks.

6. In about three weeks after this patient died, and after a heavy rain Hal C., male, aged sixteen, who lived just about one hundred and fifty yards from where patient No. 5 died, developed a well-marked case of typhoid. Almost every symptom, even to the rash, was present. He had frequent nose bleeds. Temperature reached normal on the twelfth day of treatment.

7. Just across the street, and a narrow street at that, a child near three years old developed typhoid. In all my experience I have never met with such a child. To give it medicine was to drench it like a horse. No coaxing, persuasion, or force could ever conquer it. We succeeded in getting a dose or two of calomel, one or two Woodbridge tablets, and a little elixir of lactopeptine down it. Every attempt to give it medicine was such an unpleasant procedure that the parents suggested to discontinue the attempts, and I readily assented. This child went on with well-marked typhoid symptoms and fever for about four weeks.

8. Soon after this child recovered Mrs. W., its mother, aged twenty-eight, developed well-marked typhoid. Temperature normal on the twelfth day of treatment.

9. Adjoining lot to Mrs. W., a child, aged four years, developed typhoid. The symptoms were well-marked. Temperature normal on the ninth day of treatment.

Cases numbers 10, 11 and 12, were all colored. Lived on adjacent lots and in a locality where typhoid was frequently found· Their temperatures reached normal on the 10th, 9th, and 13th days respectively.

The four remaining cases were isolated.

13. A little girl, aged eight years, lived at a place where it is said that no family has ever lived without some member of it being stricken with typhoid fever. Her case was well-marked, and during the second week she developed the most extensive and pronounced typhoid rash I have ever seen. It was not scarlet fever, but the rose-colored rash spoken of by the text-books often, and so seldom seen in practice. She was a very sick child when I first saw her and had been for two or three days before and so remained for a week; but the temperature touched normal on the thirteenth day of treatment.

14. Mrs. W., aged 42. This is the only case reported where I at all doubt the diagnosis. She began feeling badly, had elevation of temperature daily, was nauseated and jaundiced. Bowels were tender on pressure and slightly tympanitic. Temperature reached normal on the eighth day of treatment.

15. Maurice W., aged 22. Diagnosis not plain at first, so began the use of both quinine and Woodbridge tablets. After a few days discontinued quinine. Temperature normal on the twelfth day of treatment. Went to see him on the fifteenth day to dismiss him and found him at the point of death. He sat on the edge of the bed to have his shirt changed, and feeling strong had gotten over the mug unaided. Getting up he felt exhausted and fell back on the bed. When I reached him he was near death's door from heart-failure. For fourteen hours I remained with him, and can truthfully say that I have never seen any one come as near crossing the river of Jordan without doing so as did this young man. Twice I counted him as good as dead, but he finally rallied. I found he had some albuminuria and he was anemic. He never had any more elevation of temperature and except for a thrombosis of the femoral vein he would have made a nice recovery.

16. Miss W., aged 25. This was a patient who, when I first saw her, needed the service of a minister rather than that of a physician. She had been nursing a sister and brother-in-law who were in bed nine weeks from typhoid fever. Had felt badly for a week but kept up. Rode home seventeen miles through the country, facing a cold east wind and drizzle. During ride and remainder of day and night bowels moved nineteen times. When I saw her she was very weak. Pulse dicrotic; temperature $104\frac{3}{4}$. Began the use of Woodbridge tablets No. 2 every hour with a beneficial effect as regards diarrhea. The antiseptic and cholagogue effect of the tablets checked diarrhea rather than increased it. She had only four movements in the next twenty-four hours, but pulse became very weak. The third day she became comatose and took medicine badly. No Woodbridge used after second day. Taking some stimulants, but little nourishment, she lingered until the fifth day after I saw her, when she died.

In making up statistics I know it is very hard to do it fairly, especially if one wishes to draw conclusions to suit one's self. If one wishes certain statistics to appear good it is easy to find some excuse for leaving out the bad cases. If one wishes certain statistics to appear to disadvantage it is easy to count all the mild cases as errors in diagnosis. But according to the facts in the two cases here reported which died, I do not believe it justice to put them in as treated by the Woodbridge method. In fact, on account of a very unfortunate combination of circumstances, as unfortunate as ever occurred in my medical experience, they were not treated by any method at all to speak of. Had these two patients been seen a week earlier and the diagnosis made, who knows but what they might have recovered?

Of the thirteen cases which recovered and which received the Woodbridge treatment throughout, I find that the average time in which there was an elevation of temperature is eleven and a quarter days. The child which recovered without being treated had an elevation of temperature for twenty-five days.

It must not be imagined by my hearers that these are a collection of mild cases selected for a purpose. They are the cases as they came in a year's practice. The most of them were well-marked, and there was nothing in their symptoms to indicate that they might not be ill for six weeks instead of two. Cases numbers one, three, four, six, twelve and thirteen were exceedingly typical cases, with high temperatures during the first week of their illness. They were as sick as any cases of typhoid I have ever seen for the first seven or eight days. Their symptoms were so aggravated, indeed, that I felt these cases would put the treatment to a supreme test. I looked on with fear and trembling, and found little grounds for encouragement in these special cases until the beginning of the second week. When the improvement began it was remarkable and rapid.

Now as to treatment, I have never carried out Dr. Woodbridge's directions fully in any case. I seldom now use the No. 1 tablets. Upon seeing the patient and making the diagnosis or even suspecting typhoid, I give a good dose of calomel, or not, just as my judgment may direct. Then I begin with the No. 2 tablets, giving them about every half hour for the first twenty-four hours, having them swallowed with plenty of water. After twenty-four hours, if the patient has had free evacuations, I do not give them so often, but continue them throughout the course of the disease, every one, two, or three hours as needed to give the patient from two to four movements from the bowels every twenty-four hours. I seldom use the No. 3 capsules until the temperature begins to run low, and even then never cease giving the No. 2 tablets altogether until the temperature is normal and has remained so for a few days.

In addition to the above I give the following prescription in almost every case, viz.:

℞ Tr. nucis vomici ....................................ʒij

Listerin ....................................................ʒiv

Elixir lactopeptine....................................℥ij

Aquæ q. s. .................................................℥iv

M. Sig. Teaspoonful every four hours in a little water.

I do not claim much for the prescription except as a stomachic tonic, and it has, I think, a good mental effect upon the patient. It makes him think you are sufficiently interested in his case to give him more than a little yellow tablet every hour or so. Every night I have the patient sponged off with lukewarm water and gently rubbed.

For the temperature I use but little. During the first week I use small doses of phenacetin and occasional cold spongings. In the second week I use nothing, for during the past five years I have seldom found it necessary. I allow the patient to take cracked ice when desired, especially if the illness occurs in the summer months.

The diet I confine principally to cool or iced milk.

During five years' experience with the treatment outlined above I have not lost a case of typhoid except the two mentioned above; and in no case where the treatment was carried out have I seen an elevation of temperature of more than one-half or one degree after the fourteenth day of treatment. I do not claim that this method will absolutely cure every case of typhoid fever in fourteen days, or even at all. We do not always know the condition of the gut when the treatment is begun, but begun in time the failures are to be a subsequent experience with me.

I had the pleasure of having Dr. M. F. Carson, of Griffin, to see four or five of the above reported cases, and he agreed with me in the diagnosis.

